# Serum deprivation-response protein induces apoptosis in hepatocellular carcinoma through ASK1-JNK/p38 MAPK pathways

**DOI:** 10.1038/s41419-021-03711-x

**Published:** 2021-04-30

**Authors:** Xi Chen, Weijie Ma, Ye Yao, Qi Zhang, Jinghua Li, Xiaoling Wu, Chengjie Mei, Xiang Jiang, Yiran Chen, Ganggang Wang, Kunlei Wang, Yingyi Liu, Yonghua Guo, Zhisu Liu, Yufeng Yuan

**Affiliations:** 1 Department of Hepatobiliary and Pancreatic Surgery, Zhongnan Hospital of Wuhan University, Wuhan, 430071 Hubei China; 2 Department of General Medicine, Renmin Hospital of Wuhan University, Wuhan, 430071 Hubei China

**Keywords:** Tumour biomarkers, Tumour-suppressor proteins

## Abstract

Serum deprivation-response protein (SDPR), a phosphatidylserine-binding protein, which is known to have a promising role in caveolar biogenesis and morphology. However, its function in hepatocellular carcinoma (HCC) was still largely unknown. In this study, we discussed the characterization and identification of SDPR, and to present it as a novel apoptosis candidate in the incidence of HCC. We identified 81 HCC cases with lower SDPR expression in the tumor tissues with the help of qRT-PCR assay, and lower SDPR expression was potentially associated with poor prognostication. The phenotypic assays revealed that cell proliferation, invasion, and migration were profoundly connected with SDPR, both in vivo and in vitro. The data obtained from the gene set enrichment analysis (GSEA) carried out on the liver hepatocellular carcinoma (LIHC), and also The Cancer Genome Atlas (TCGA) findings indicated that SDPR was involved in apoptosis and flow cytometry experiments further confirmed this. Furthermore, we identified the interaction between SDPR and apoptosis signal-regulating kinase 1 (ASK1), which facilitated the ASK1 N-terminus-mediated dimerization and increased ASK1-mediated signaling, thereby activating the JNK/p38 mitogen-activated protein kinases (MAPKs) and finally enhanced cell apoptosis. Overall, this work identified SDPR as a tumor suppressor, because it promoted apoptosis by activating ASK1-JNK/p38 MAPK pathways in HCC.

## Introduction

Hepatocellular carcinoma (HCC) has been identified as the most common type of primary liver cancers, the latest epidemiological studies showed that HCC accounted for the third largest number of tumor-related deaths. During 2018, about 840,000 new cases of liver cancer have been reported worldwide, which caused 780,000 deaths, approximately^1^. Despite a series of preventative and treatment measures that have been taken, HCC still remains the third leading cause of deaths that occurs from cancer in China, it also stands fourth among the common malignancy incidents in the same region^2^. Although several treatment procedures have been applied for HCC, the rate of recurrence is potentially high, and prognostication of HCC patients still remains very poor^3^. It is essential to identify pivotal antitumor molecules and establish potential therapeutic targets.

As a main type of programmed cell death^4^, apoptosis has been appeared to regulate cell proliferation and death balance, disruption of the balance may result in uncontrolled cell proliferation and eventually cancer or degenerative disorders^5^. The deregulation of cell death process is associated with aberrant cell proliferation, leading to growth of cancer and drug resistance^6^. As a major hallmark of tumorigenesis, apoptosis is considered to be one of the most promising antitumor mechanisms in cancer therapy^7^. However, treatment resistance still remains a major issue during the course of HCC incidence as effective interventions have not been fully discovered. Therefore, it is necessary to better understand the mechanisms of apoptosis and develop more effective molecular-targeted therapies in order to overcome treatment resistance of HCC.

Here, we reported a novel tumor suppressor, serum deprivation-response protein (SDPR) (also called cavin-2), in inducing HCC cell apoptosis. SDPR was firstly identified as a phosphatidylserine-binding protein, as a member of cavin family, SDPR has been studied on the role of regulating caveolae formation and inducing membrane curvature^8^, but the potential function in cancer was rarely described previously. However, evidence from a recent study showed that SDPR was a metastasis suppressor through inhibiting epithelial–mesenchymal transition (EMT), migration and promoting apoptosis by relieving the inhibition of the extracellular-signal-regulated kinase (ERK) pathway in breast tumor^9^. A more recent study indicated that SDPR served as a key intermediary agent in miR-577-ERK-NF-κB pathway positive-feedback loop which regulated gastric cancer progression^10^. In addition, the study reported the lower expression of SDPR in the cancer of kidney, breast, and prostate^11^ and HCC^12^. Nevertheless, its specific functions and mechanisms in HCC still remains undiscovered.

The present study was conducted to identify the potential role of SDPR in the development and progression of HCC. We found that SDPR expression was markedly associated with the HCC cells’ malignant phenotypes and prognostication of HCC patients. Mechanically, SDPR induced cells apoptosis by promoting ASK1 N-terminus-mediated dimerization and activating ASK1-JNK/p38 MAPK pathways in HCC.

## Materials and methods

### Cell culture

The human HCC cell lines, HCCLM3, Huh-7, Hep3B, MHCC97L, HepG2, and SK-Hep1, and normal liver cell line L02 were obtained from the Cell Bank, Type Culture Collection, Chinese Academy of Sciences (CBTCCCAS, Shanghai, China). Cell lines were authenticated by short tandem repeat profiling. Total cells were cultivated in the Dulbecco’s modified Eagle’s medium (DMEM) (Gibco, CA, USA) added with 10% fetal bovine serum (FBS) (Tico Europe, Netherlands), in a humidified atmosphere of 5% CO_2_ at 37 °C. To avoid microbial contamination, culture medium was also added with 1% penicillin–streptomycin (Gibco, CA, USA).

### Clinical specimens

In this study, all those patients were included, who had been diagnosed with HCC from 2016 to 2020, who underwent hepatectomy in the Zhongnan hospital of Wuhan University of China, and those who did not receive any chemotherapy or radiation therapy before. Paired tissue specimens (cancer and normal tissues) were histologically confirmed by competent pathologists, collected within 30 min after the surgery, and were preserved in liquid nitrogen till further processing. All patients included were informed and approved the study.

### RNA extraction and quantitative real-time polymerase chain reaction (qRT-PCR) analysis

Using a Trizol reagent kit (Invitrogen, California, USA) with regard to the manufacturer’s instructions, total RNA was extracted from the tissues as well as cell lines. To determine the concentration and purity of RNA, the NanoDrop ND2000 instrument (Thermo Scientific, Massachusetts, USA) was employed. Reverse transcription experiments were performed using HiScript II Q RT SuperMix (Vazyme, Nanjing, China) and then cDNA was obtained. The CFX96TM Real-Time System (Bio-Rad) was used to perform the QRT-PCR, and the level of the mRNA expression was assessed with the help of the comparative cycle threshold (Ct) method (2^−ΔCt^). All experiments were performed in triplicate, the sequences of the primers used were listed in Supplementary Table 1.

### Western blotting analysis

The BCA Protein Assay Kit (Thermo Scientific) was utilized to qualify the Cell Lysis solution. Proteins were transferred to polyvinylidene fluoride after being separated on 10% sodium dodecyl sulfate-polyacrylamide gel electrophoresis (SDS-PAGE). In the next step, membranes were blocked with skim milk powder in TBST for 1 h and then incubated overnight at 4 °C with specific primary antibodies. Subsequently, membranes were added with the HRP-labeled antibody, then incubated at room temperature (RT) for a period of 2 h. Utilizing the ECL chemiluminescence reagent (Bio-Rad), antibody-bound proteins were identified. The information of antibodies utilized in this study were displayed in Supplementary Table 2.

### Immunohistochemistry (IHC)

Slides were prepared with 4 μm thick paraffin sections, then xylene and ethanol were used to deparaffinized rehydrate the slides, respectively. However, 0.3% hydrogen peroxide was utilized for the inactivation of endogenous peroxidase. While following the manufacturer’s protocol, all of the necessary steps were performed by employing an UltraSensitiveTM S-P kit (Maixin, Fuzhou, China).

### Plasmid construction, Lentiviral construction and cell transfections

Full-length (FL) or truncated human SDPR and ASK1 gene sequences were amplified by PCR and cloned into phage-FLAG/HA/MYC vectors. The vector and lentivirus were transfected and SDPR was stably expressed in HCC cell lines. The small interfering RNA (siRNA) targeting SDPR were obtained from GENECREATE (Wuhan, China), then the transfection of HCC cells was accomplished with the help of SDPR-siRNA by utilizing the GenMute (SignaGen, Maryland, USA) in accordance with the manufacturer’s protocol. Moreover, total RNA and protein were extracted after 24 h of the transfection process. The sequences of siRNA have been displayed in Supplementary Table 3.

### Cell proliferation assays

Cell proliferation was detected by using the colony formation assay and cell counting kit-8 (CCK-8). To perform CCK-8 assay, cells were first sown into 96-well plates at a density of 5 × 10^3^ cells/well and were kept for incubation in a complete culture medium. Every single well was added with 10 μl CCK-8 solution (Dojindo, Kumamoto Ken, Japan), and cells were incubated for a duration of 2 h. In the next step, absorbance was measured at 450 nm with the help of a microplate reader. Clonogenic assay was performed by seeding 1000–2000 cells per 6-well plate and were grown for a period of 2 weeks. The desired clones were stabilized with 4% paraformaldehyde, stained with 0.1% crystal violet solution, and counted under the light microscope.

### Wound healing assay

HCC cells were sown in a 6-well plate at a density of 1 × 10^6^ cells per well, and cultured without FBS for 24 h in DMEM. Wounds were created with the help of a 100-μl plastic pipette tip. Imaged of the scratches were captured at 0 and 24, 48, or 72 h. The digital pictures were used to calculate the areas of scratch without cells and the remaining areas.

### Transwell assay

Transwell invasion was conducted in 24-well transwell plates, which possess 8-μl pore-size Matrigel-coated chambers. In the serum-free medium on the upper chamber, a sum of 1 × 10^5^ cells were sown, however, the medium with 10% FBS was placed in the lower chamber. The cells holding the upper chamber were removed after a complete incubation of 24–72 h, and the invaded cells were stabilized and stained with 4% paraformaldehyde and 0.1% crystal violet solution, respectively.

### Cell apoptosis analysis

Cell apoptosis was detected by FC500 flow cytometer (Beckman-Coulter), with regard to the manufacture’s protocol of an Annexin V-fluorescein isothiocyanate (FITC) apoptosis detection kit (Beyotime, Shanghai, China).

### In vivo experiments

The Central Laboratory of Animal Science located at Wuhan University (Wuhan, China), provided us with male athymic 5-week-old BALB/c nude mice, which were raised in a particular facility free from any pathogen. the mice were randomly assigned for the experiments using a table of random numbers. The sample size is estimated to effectively detect a significant difference among the groups. Investigators were blinded to the group allocation during the experiment. To evaluate the tumor proliferation in vivo, HCCLM3 cells (5 × 10^6^) stably transfected with SDPR or negative control were suspended in PBS and then administered with direct injection into the armpit of 5 mice, respectively. Tumor size was measured with a vernier caliper. Mice were lost after 4 weeks, and tumors were dissected and imaged. Animals used in the experimental work of this study were treated humanely, with regard to the National Institutes of Health guide for the care and use of Laboratory animals (NIH Publications No. 8023, revised 1978). The study of animal was approved by the Ethics Committee of Zhongnan Hospital.

### Endogenous co-immunoprecipitation (Co-IP) and exogenous Co-IP

Huh-7 cells transfected with Flag-tagged SDPR were cultured in 10-cm dishes and lysed in immunoprecipitation buffer (20 mM Tris-HCl, pH 7.4; 150 mM NaCl; 1 mM EDTA, pH 8.0; 1% NP-40; 1× Protease and Phosphatase Inhibitor Cocktail). Then 1 μl FLAG antibody or IgG and 10 μl of Protein A/G magnetic beads (pre-washed with lysis buffer) were introduced into the cell lysate, and were incubated for 3 h at 4 °C. In the following step, the beads were sequentially rinsed two times with high salt and low salt wash buffer. Finally, proteins were eluted from the beads by 2× SDS loading buffer and boiled at 95 °C for 10 min prior to SDS-PAGE and immunoblotting with FLAG and ASK1 antibodies. For Exogenous co-IP, HEK293T cells were co-transfected with FLAG/HA-tagged SDPR and ASK1, the next steps are the same as for endogenous co-IP protocol.

### Co-IP mass spectrometry (Co-IP MS) and silver staining

Huh-7 cells transfected with FLAG-SDPR were cultured in 10-cm dishes, following Co-IP as described previously. Proteins were analyzed using SDS-PAGE, and silver staining was carried out by utilizing Pierce Silver Stain Kit (Thermo Scientific, Massachusetts, USA) with regard to the manufacturer’s recommendations. Then gels were subjected to mass spectrometry-based analysis.

### Immunofluorescence (IF)

Cells grown on coverslips were washed three times with phosphate-buffered saline (PBS), and then paraformaldehyde was utilized to fix them for 15 min. Cells were blocked with 5% bovine serum albumin for a duration of 30 min after being permeabilized with 0.5% NP-40 for 20 min. In the following steps cells along with primary antibody were incubated for 2 h at RT, and subsequently incubated with fluorescein conjugated secondary antibody for 1 h. Finally, the DAPI and confocal microscopy were used to stain and image the slides, respectively.

### Statistical analysis

GraphPad Prism 8.0 software and SPSS 21.0 software was used to carry out the Statistical analyses. Using the Student’s *t* test and *χ*^2^ as appropriate, parametric statistical analysis was conducted. For grouping, the median SDPR expression was considered as a cut-off value. A low SDPR group was defined as a value lower than the 50th percentile, in each of the 81 patients. However, a high SDPR group was defined as a value greater than the 50th percentile, in each of 81 patients. Following the Kaplan-Meier methodology, the survival curves were plotted. The entire values have been shown as the mean ± SD. *P* values were specified as follows: *P < 0.05; **P < 0.01; ***P < 0.001.

## Results

### SDPR was significantly downregulated in HCC and was associated with HCC prognosis

**Fig. 1:**
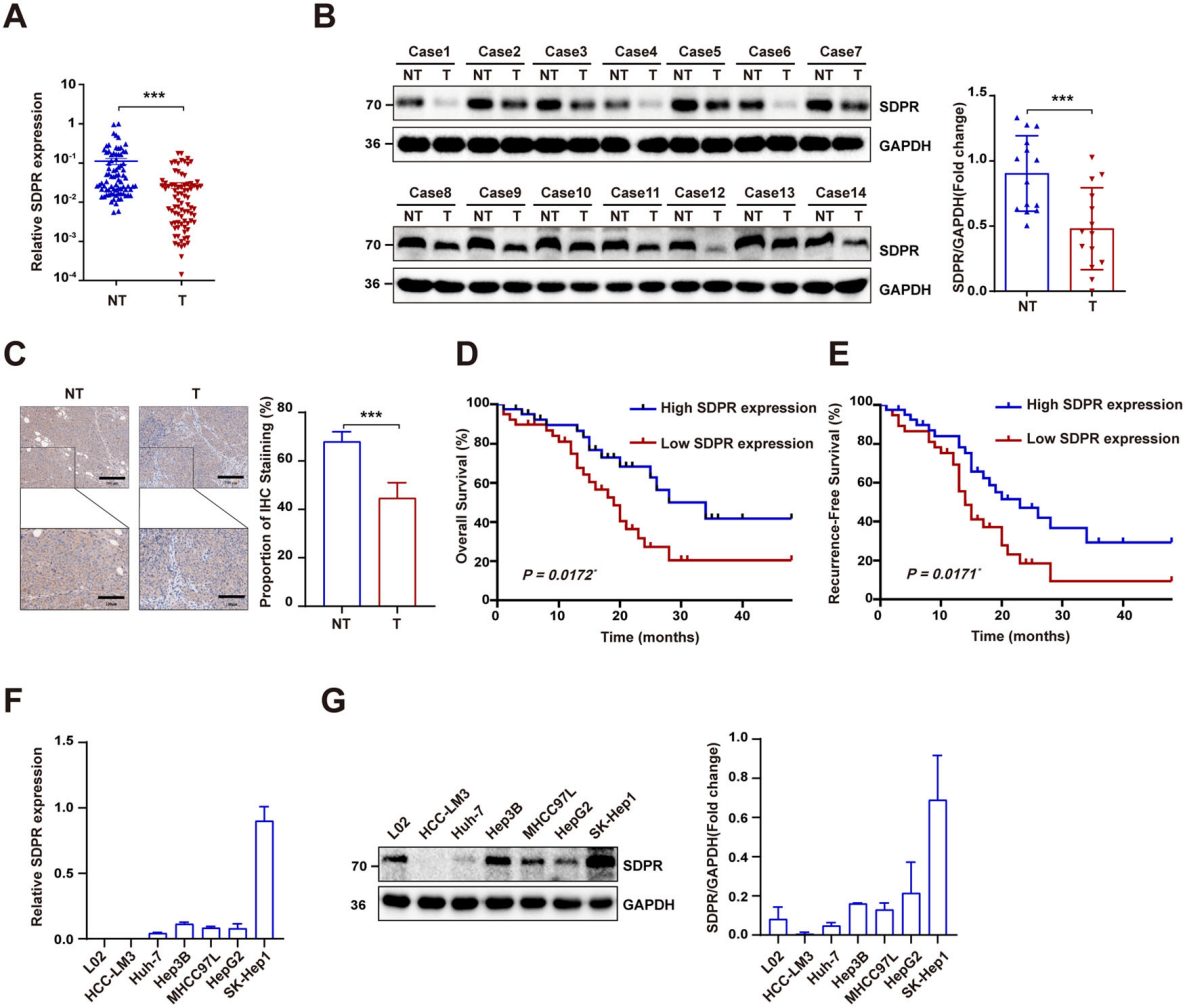
SDPR expression was reduced in HCC and predicted poor prognosis. **A** SDPR expression was downregulated in HCC tissues compared to non-tumor tissues, identified by the qRT-PCR analyses of HCC samples of 81 patients. **B** The level of SDPR protein was observed in 14 sets of human HCC cancers tissues and peritumor tissues. **C** SDPR immunohistochemistry (IHC) stain in clinically identified HCC tumor samples and adjacent non-tumor samples. **D**, **E** Kaplan–Meier analysis of overall survival (OS) and recurrence free survival (RFS) in 81 HCC patients from our hospital. **F**, **G** Relative SDPR mRNA and protein expression level in six human HCC cell lines: HCCLM3, Huh-7, Hep3B, MHCC97L, HepG2, SK-Hep1, and normal liver cell L02. *p < 0.05; ***p < 0.01; ***p < 0.001.

To identify the function of SDPR, the SDPR expression profile was accessed by Western blot and qRT-PCR. SDPR mRNA expression was detected in 81 HCC patients between tumor and adjacent non-tumor tissues by employing quantitative RT-PCR. The results revealed a considerably lower expression of SDPR mRNA in HCC tissues compared to peritumor tissues (Fig. 1A). Moreover, the Western blot and IHC analyses confirmed the reduced protein expression of HCC tissues (Fig. 1B, C). Clinical features of patients were presented in Table 1, SDPR mRNA expression were significantly associated with TNM stage, tumor size, PVTT, and histologic grade. More importantly, Kaplan–Meier analysis indicated that low SDPR expression was evidently associated with poor overall survival and recurrence free survival (Fig. 1D, E). As shown in Fig. 1F, G, the SDPR expression was found profoundly upregulated in SK-Hep1 and downregulated in HCCLM3 and Huh-7 cell lines.

**Table 1:** Clinical characteristics of 81 HCC patients according to SDPR expression level.

Characteristics	Number of cases	SDPR expression		*p* Value
		Low (*n* = 40)	High (*n* = 41)	
*Age (years)*				0.483
≥Median (58)	41	16	25	
<Median (58)	40	14	16	
*Gender*				0.171
Male	64	34	30	
Female	17	6	11	
*Tumor size*				0.029*
≥5 cm	56	22	34	
<5 cm	25	18	7	
*Histologic grade*				0.001***
Poor or moderate	44	15	19	
Well	37	25	12	
*HBV infection*				0.807
Positive	67	33	34	
Negative	14	7	7	
*Liver cirrhosis*				0.512
Yes	51	24	26	
No	30	16	14	
*Serum AFP (μg/L)*				0.332
≥400	59	30	29	
<400	22	10	12	
*PVTT*				0.015*
Yes	28	10	18	
No	53	30	23	
*TNM stage*				0.006**
Stage III + IV	47	16	31	
Stage I + II	34	24	10	

*PVTT* portal vein tumor thrombus.

*p < 0.05; **p < 0.01; ***p < 0.001.

The *p* values with significance are marked in bold.

### SDPR overexpression leaded to decreased proliferation, invasion, and migration of hepatoma cells in vitro and restrained tumor growth in vivo

**Fig. 2:**
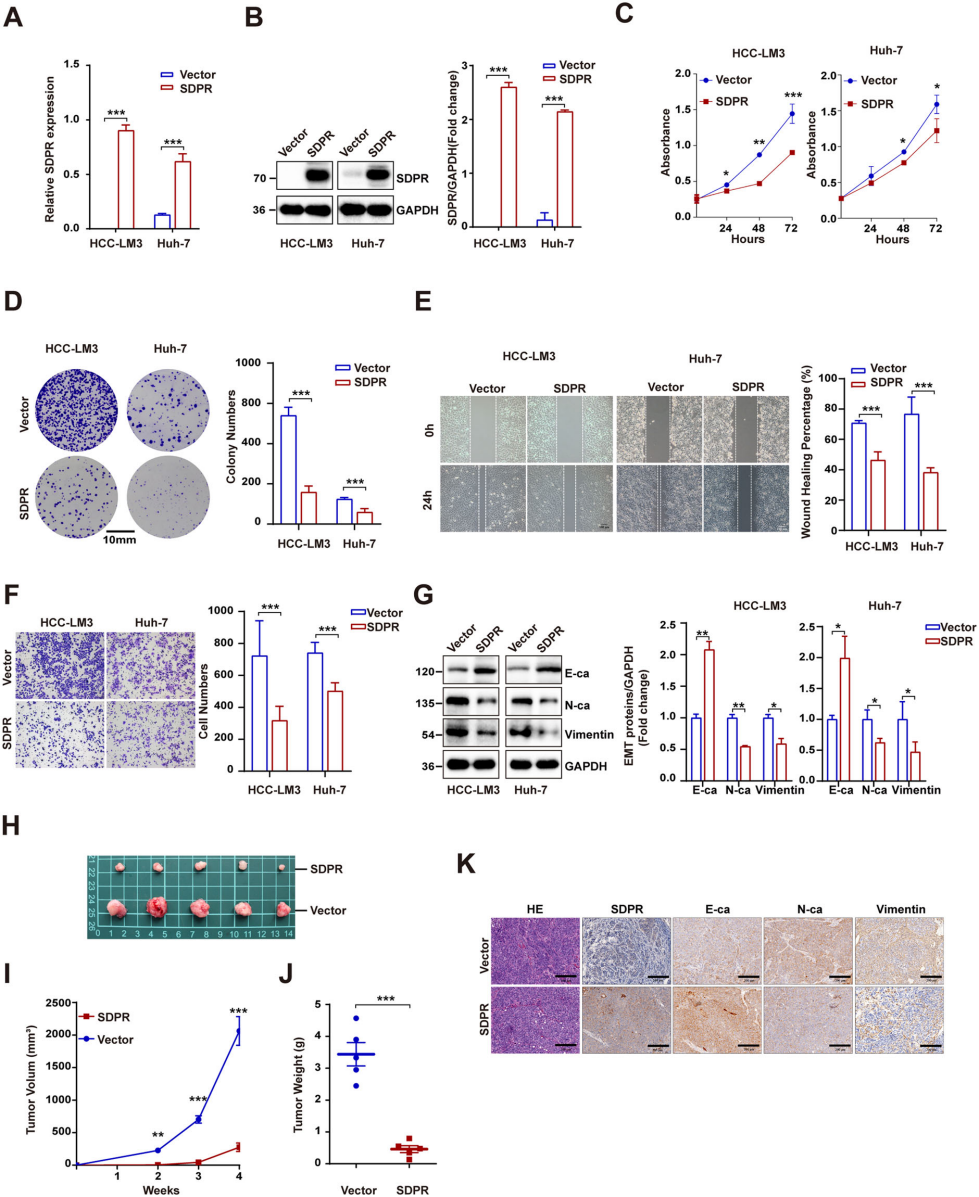
SDPR overexpression inhibited HCC cells proliferation, migration, and invasion in vitro and repressed tumor growth in vivo. **A**, **B** Identification of cell lines stably integrating SDPR by qRT-PCR and western blotting in HCCLM3 and Huh-7 cell lines. **C**, **D** Cell counting kit-8 analyses and colony formation revealed that SDPR restrained hepatoma cells growth in SDPR overexpressing HCCLM3 and Huh-7 cell lines. **E**, **F** Overexpression of SDPR in HCCLM3 and Huh-7 cell lines suppressed cell migration and invasion and assessed by wound-healing and transwell analyses. **G** The EMT proteins level of E-cadherin (E-Ca), N-cadherin (N-Ca), and vimentin in HCCLM3 and Huh-7 cell lines after SDPR overexpressed. **H** Images of tumors obtained from nude mice were implanted with up-regulated SDPR expression HCCLM3 cells (*n* = 5) or control cells (*n* = 5). **I**, **J** Tumor growth curves and tumor weights of SDPR group and control. **K** Images that indicate the hematoxylin and eosin (H&E) and IHC staining features of E-cadherin, N-cadherin, and vimentin in tumor xenografts of nude mice in SDPR group and control group. *p < 0.05; **p < 0.01; ***p < 0.001.

To elucidate the antitumor role of SDPR, we firstly generated HCCLM3 and Huh-7 cell lines stably integrating SDPR. SDPR-overexpressed stable cell lines were shown in Fig. 2A, B by qRT-PCR and western blotting. SDPR overexpression significantly repressed the growth of HCC cells according to CCK-8 assay (Fig. 2C), colony formation assay further conformed that SDPR decreased HCC cells proliferation (Fig. 2D). Moreover, wound-healing assay and transwell assay revealed that SDPR overexpression reduced the migration (Fig. 2E) and invasion (Fig. 2F) of HCC cells, when compared with the control. To illustrate the influence of SDPR overexpression on EMT of HCC cells, we detected the expression of E-cadherin, N-cadherin, and vimentin by western blotting. The results indicated that E-cadherin was upregulated while N-cadherin and vimentin were downregulated in SDPR-overexpressed stable cell lines (Fig. 2G). The antitumor effect of SDPR on HCC cells was investigated in vivo, by injecting the SDPR-overexpressed HCCLM3 cells and normal HCCLM3 cells, subcutaneously into nude mice. The SDPR group was found with profoundly lower volumes and weights of the tumors than those in the control group (Fig. 2H–J). In addition, IHC staining indicated increased expression of E-cadherin, while N-cadherin and vimentin were downregulated in SDPR-overexpressed HCCLM3 tumors (Fig. 2K).

### The silence of SDPR increased proliferation, invasion and migration of HCC and normal liver cells

**Fig. 3:**
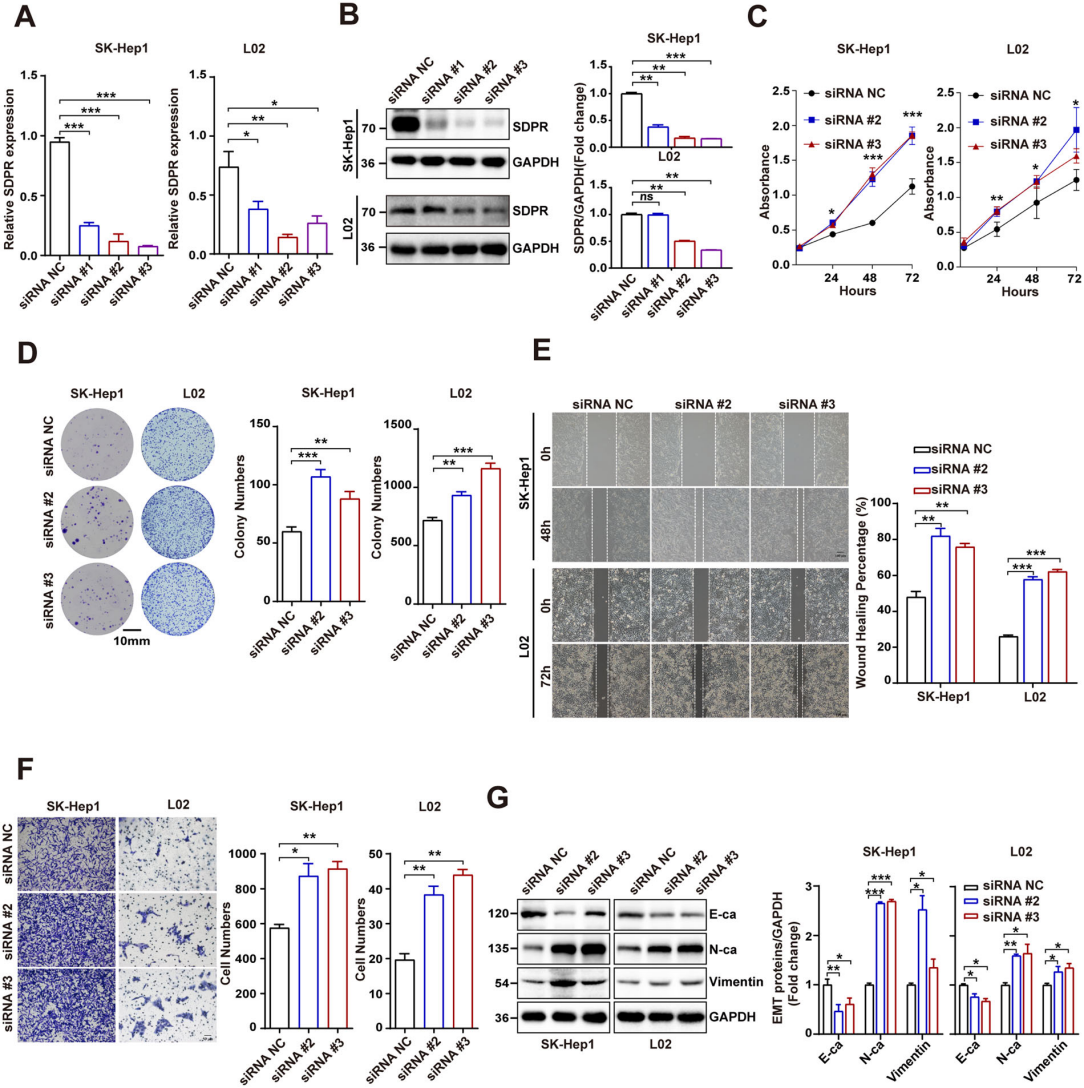
Depletion of SDPR promoted cell proliferation, migration, and invasion of SK-Hep1 and L02 cells. **A**, **B** SDPR knockdown efficiency was identified by western blotting and qRT-PCR. **C**, **D** CCK-8, and colony formation assays showed that SDPR knockdown facilitated SK-Hep1 and L02 cells growth. **E**, **F** Depletion of SDPR promoted cell invasion and migration by transwell and wound-healing assays. **G** The EMT proteins level of E-cadherin (E-Ca), N-cadherin (N-Ca), and vimentin in SDPR depleted SK-Hep1 and L02 cell lines. *p < 0.05; ***p < 0.01; ***p < 0.001.

To further clarify the function of SDPR in HCC cells, we designed three small interfering RNAs (siRNAs). It has been displayed in Fig. 3A, B that the SDPR expression in SK-hep1 and L02 cells was significantly reduced in three siRNA groups, among which siRNA #2 and #3 produced the best reduction of SDPR and were chosen for the following functional experiments. The depletion of SDPR significantly promoted the cells growth and proliferation by CCK-8 and colony formation assays (Fig. 3C, D) and facilitated cells migration and invasion by wound-healing assay and transwell assay (Fig. 3E, F). In addition, Western blotting indicated that the expression of N-cadherin and vimentin increased while E-cadherin decreased in SDPR-depleted SK-Hep1 and L02 cells (Fig. 3G).

### SDPR activated apoptosis signaling pathway and leaded to increased apoptosis of HCC cells

**Fig. 4:**
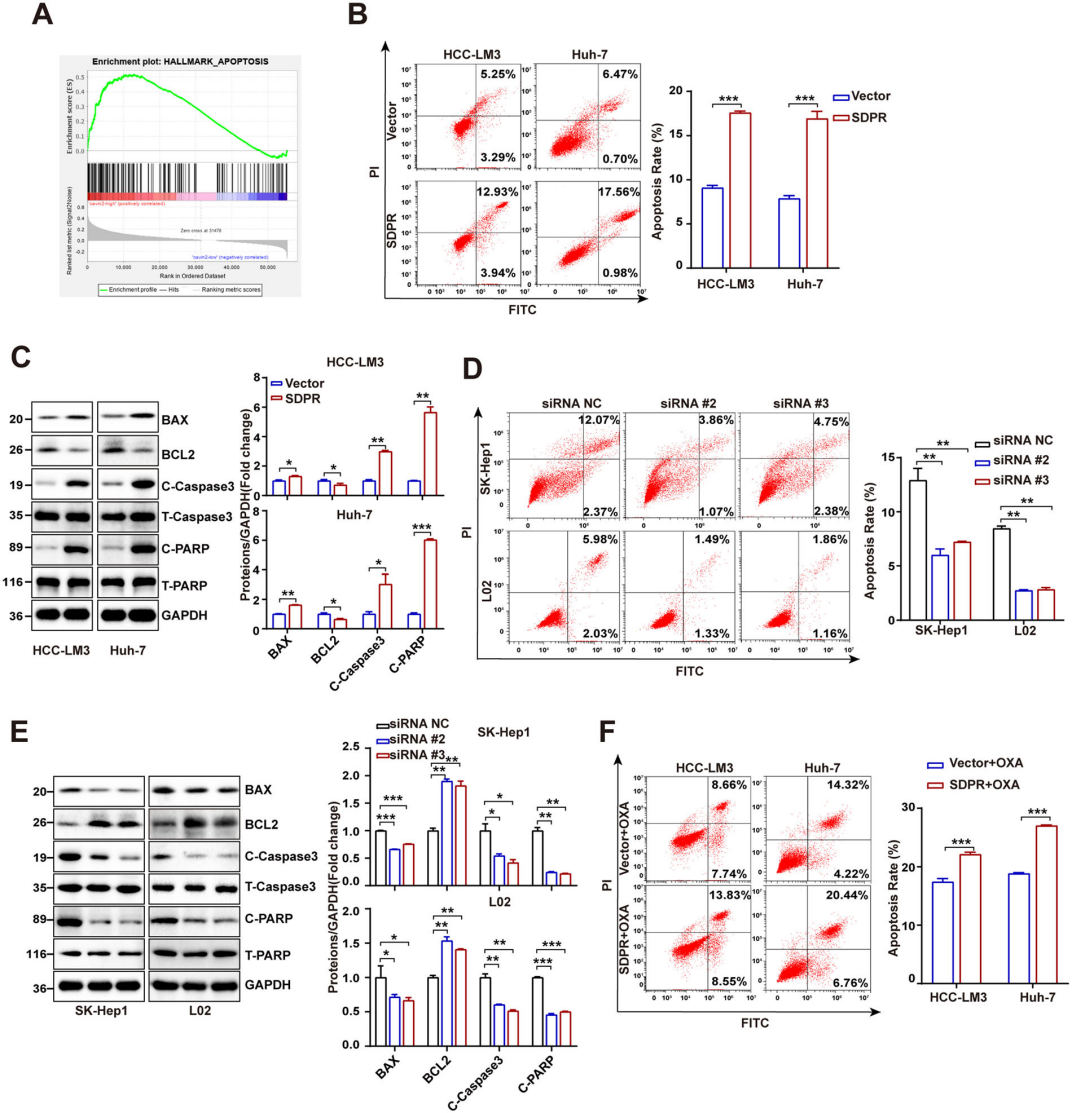
SDPR activated apoptosis signaling pathway and induced cell apoptosis. **A** Positive enrichment for the gene signature associated apoptosis between the low and high SDPR expression groups of TCGA dataset. **B** SDPR overexpression promoted cell apoptosis in HCCLM3 and Huh-7 cell lines. **C** The changes of apoptosis-related proteins in SDPR overexpressing HCCLM3 and Huh-7 cell lines. **D** SDPR-depletion inhibited cell apoptosis in SK-Hep1 and L02 cell lines. **E** SDPR knockdown changed the level of apoptosis-related proteins. **F** SDPR significantly promoted OXA-induced HCC cell apoptosis. *p < 0.05; ***p < 0.01; ***p < 0.001.

To evaluate the antitumor role of SDPR and its underlying mechanism in HCC, gene set enrichment analysis (GSEA) was performed on the gene data from TCGA (The Cancer Genome Atlas) database (https://portal.gdc.cancer.gov/) using the TCGA software 4.0.1. The single gene GSEA analysis showed positive enrichment for the gene signature associated apoptosis between the low and high heterogeneity groups (Fig. 4A). Therefore, considering the flow cytometry, cell apoptosis was detected. The results revealed that the apoptotic rate of SDPR overexpressed HCCLM3 and Huh-7 were increased compared with the vector groups (Fig. 4B). Moreover, the antiapoptotic protein’s (Bcl-2) expression was downregulated, while the apoptotic protein markers, including Bax, Cleaved-caspase3 and Cleaved-PARP, were increased in SDPR overexpressed groups (Fig. 4C). On the contrary, SDPR-depletion inhibited cell apoptosis and leaded to opposite changes of apoptosis-related proteins (Fig. 4D, E) in SK-Hep1 and L02 cell lines. Oxaliplatin (OXA) is a kind of platinum-deprived chemotherapeutic drug which serves as a tumor suppresser by inducing apoptosis, but dispalyed poor chemotherapy efficacy in HCC because of chemotherapy resistance^13,14^. To further understand the promising therapy effect of SDPR in HCC, OXA chemotherapy experiments were performed. SDPR overexpressed HCCLM3 and Huh-7 and control groups were havered with 5 mg/L OXA for 12 h, then the flow cytometry results showed that SDPR significantly promoted OXA-induced HCC cell apoptosis (Fig. 4F).

### SDPR interacted with ASK1 at N-terminal region and promoted ASK1 N-terminal dimerization

**Fig. 5:**
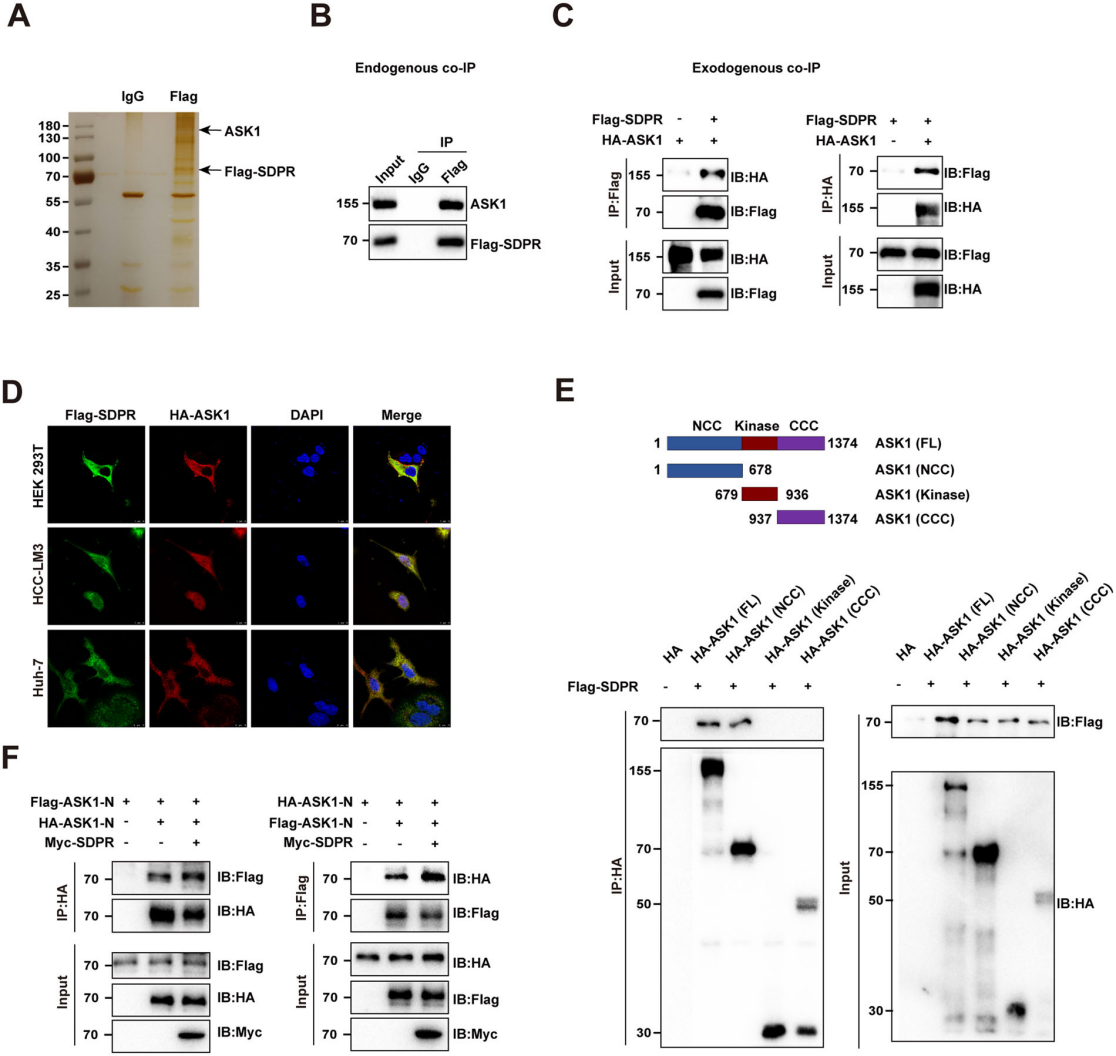
SDPR interacted with and activated ASK1. **A** SDPR-binding proteins were presented by immunoblotting and silver staining. **B**, **C** Endogenous co-IP assay and exogenous co-IP assays were conducted to find the interaction of SDPR and ASK1. **D** Immunofluorescence analysis showed SDPR and ASK1 co-located after co-transfected with FLAG-SDPR and HA-ASK1 in HEK-293T, HCCLM3, and Huh-7 cells. **E** HA-tagged full-length and three truncated mutants ((1) N-terminal, HA-ASK1^1-678^, (2) Kinase, HA-ASK1^679-936^, and (3) C-terminal, HA-ASK1^937–1374^) of ASK1 were cotransfected with FLAG-SDPR in HEK 293T cells and then lysates were immunoprecipitated by utilizing anti-HA antibody. **F** HA/FLAG-tagged ASK1 N-terminal and MYC-tagged SDPR were cotransfected into HEK 293T and immunoprecipitated using anti-HA/FLAG antibodies to detect ASK1 N-terminus dimerization. *p < 0.05; ***p < 0.01; ***p < 0.001.

Co-IP MS assay was conducted for the detection of SDPR-binding proteins, which were potentially associated with SDPR. The binding proteins were visualized by immunoblotting and silver staining (Fig. 5A). Totally, 259 kinds of protein bound to SDPR were identified by mass spectrometry. Being an important apoptosis regulator, ASK1 caught our attention. To confirm the interaction of SDPR and ASK1, a series of protein interacting assays were conducted to evaluate such PPI in different cellular environments and experimental conditions. First, endogenous co-IP assay was performed in FLAG-tagged SDPR overexpressed Huh-7 cells, results indicated that ASK1 was present in the FLAG immunocomplex, however absent in the IgG immunocomplex (Fig. 5B). Exogenous co-IP assays were followed by co-transfecting FLAG-tagged SDPR and HA-tagged ASK1 into HEK-293T cells, then coprecipitation using an anti-FLAG or an anti-HA antibody further demonstrated the PPI (Fig. 5C). Moreover, the interaction between SDPR and ASK1 could be well confirmed through immunofluorescence analysis by their co-location in HEK-293T, HCCLM3, and Huh-7 cells transfected with HA-ASK1 and with FLAG-SDPR as well (Fig. 5D). To better understand the binding region between SDPR and ASK1, we carried out co-IP of SDPR with different structural elements of ASK1. The ASK1 structure can be divided into three parts, the C-terminal coiled-coil domain (CCC), N-terminal coiled-coil domain (NCC), and a central kinase domain. We constructed three HA-tagged truncated mutants of ASK1: (1) N-terminal, HA-ASK1^1–678^, (2) Kinase, HA-ASK1^679–936^, and (3) C-terminal, HA-ASK1^937–1374^. The three ASK1 mutants and FL ASK1 were cotransfected with FLAG-tagged SDPR, respectively into HEK 293T cells, then coprecipitation using anti-HA antibody showed that SDPR were detected in FL-ASK1 and ASK1^1–678^ groups, suggesting that NCC fragment may contain the SDPR binding site (Fig. 5E). Previous study indicated that ASK1 N-terminal hemophilic, followed by autophosphorylation is potentially necessary for its stimulation^15^. Therefore, dimerization experiment was performed by transfecting HA/FLAG-tagged ASK1 N-terminal and MYC-tagged SDPR into HEK 293T and immunoprecipitated using anti-HA/FLAG antibody. The results demonstrated that SDPR facilitated the N-terminus-mediated dimerization (Fig. 5F). Overall, these findings illuminated that SDPR bound the N-terminal of ASK1 and promoted ASK1 homodimer formation.

### SDPR promoted the apoptosis of HCC cells via ASK1-JNK/p38 MAPKs signal pathway

**Fig. 6:**
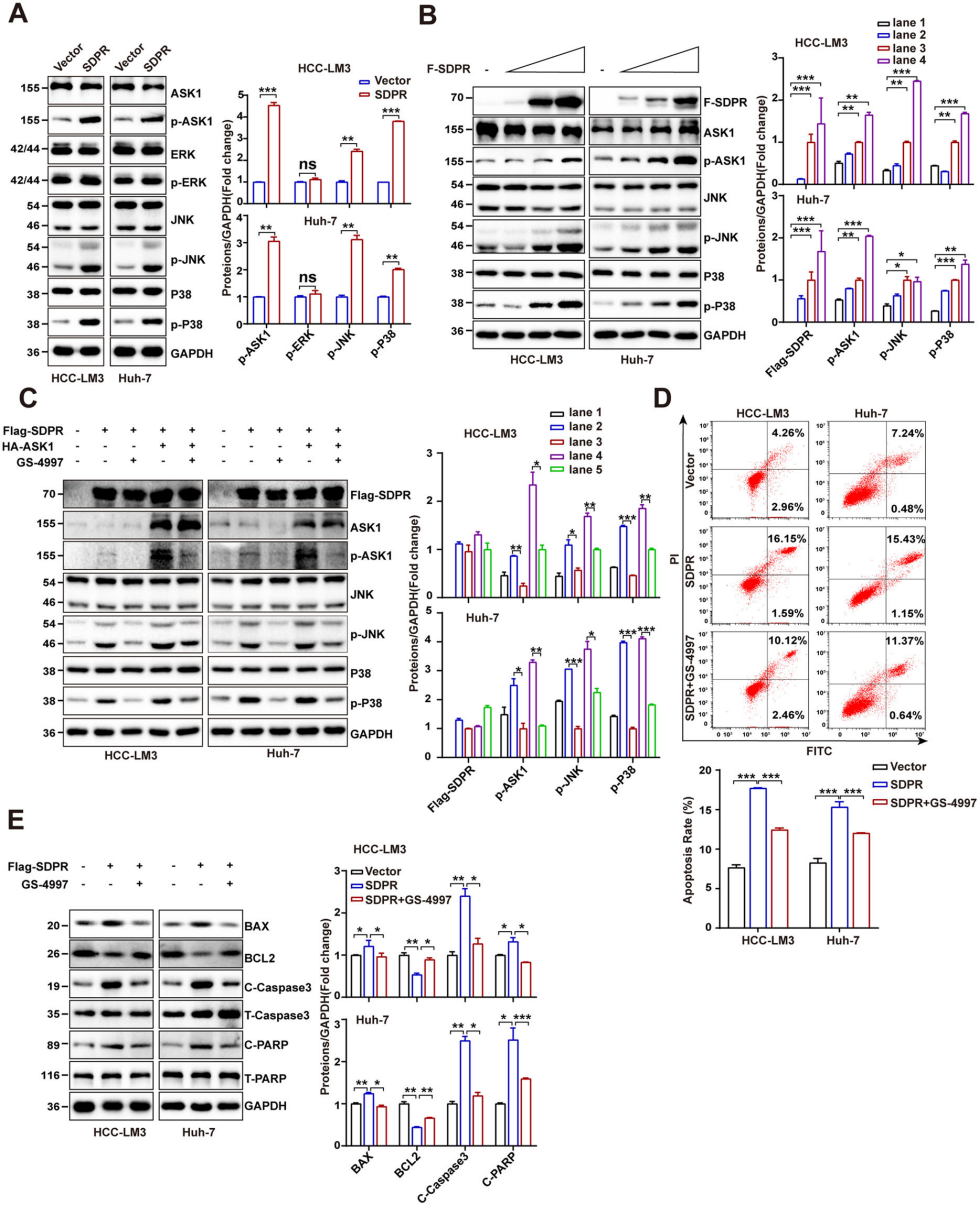
SDPR promoted the apoptosis of HCC cells via ASK1-JNK/p38 MAPKs signal pathways. **A** The levels of ASK1-MAPKs pathways key proteins, phosphorylated and total ASK1, ERK1/2, JNK1/2, and p38 in vector and SDPR overexpressing HCCLM3 and Huh-7 cells. **B** The effect of SDPR gradient transfection in HCCLM3 and Huh-7 cells on proteins level of phosphorylation and total ASK1, JNK1/2, and P38. **C** The Western blot evaluation of protein expression of phosphorylated and total ASK1, JNK1/2, and p38 in SDPR and ASK1 cotransfected treatment of Huh-7 and HCCLM3 cells with ASK1 inhibitor GS-4997. **D** FACS analysis showed GS-4997 inhibited ASK1-induced HCC apoptosis. **E** The levels of apoptotic related proteins changes after treated with GS-4997 in HCCLM3 and Huh-7 cells. *p < 0.05; ***p < 0.01; ***p < 0.001.

Based on the significant pro-apoptotic effects of SDPR and its interaction with ASK1, next, we sought out potential signaling pathways underlying its functional roles. We first examined the phosphorylation of ASK1 (Thr845) and the activation of MAPKs including ERK1/2, JNK1/2, and P38 in HCCLM3 and Huh-7 cells transfected with SDPR. Consist with expectations, samples overexpressing SDPR displayed a considerable elevation in phosphorylated ASK1. However, only the phosphorylation of JNK1/2 and P38 were increased by SDPR overexpression, whereas phosphorylated ERK1/2 remained unaffected (Fig. 6A). Subsequently, we further detected the phosphorylation of ASK1, JNK1/2, and P38 after gradient transfection with Flag-SDPR in HCCLM3 and Huh-7 cells. Phosphorylation of ASK1, JNK1/2, and P38 were shown to be directly proportional to the amount of SDPR transfection (Fig. 6B). Considering the complexity of MAPK signaling pathway, it is critical to examine whether ASK1 appeared to be the upstream kinase that acted as a mediating entity for the SDPR-evoked phosphorylation of JNK1/2 and P38. The ASK1 inhibitor, GS-4997 (Selonsertib) was utilized for the further evaluation of the ASK1 effect toward the SDPR expression. The activity of ASK1 could be inhibited by GS-4997 and the phosphorylation of JNK/P38 were further suppressed^16^. As shown in Fig. 6C, SDPR overexpression and co-expression of SDPR and ASK1 led to increases in phosphor-ASK1, as well as increases in phosphor-JNK/P38. However, the GS-4997 treatment inhibited the ASK1-JNK/P38 pathway (Fig. 6C). Based on the results above, we performed apoptosis assays on SDPR overexpressed cells. The data revealed that GS-4997 treatment significantly inhibited ASK1-induced HCC apoptosis (Fig. 6D). Furthermore, the Western blotting showed that GS-4997 reversed apoptotic related proteins changes that induced by SDPR in HCCLM3 and Huh-7 cells (Fig. 6E). Together these results suggested that SDPR acted ASK1 to promote JNK/P38 activation and induced HCC cells apoptosis.

### ASK1 inhibitor GS-4997 restored the SDPR-induced antitumor effects

**Fig. 7:**
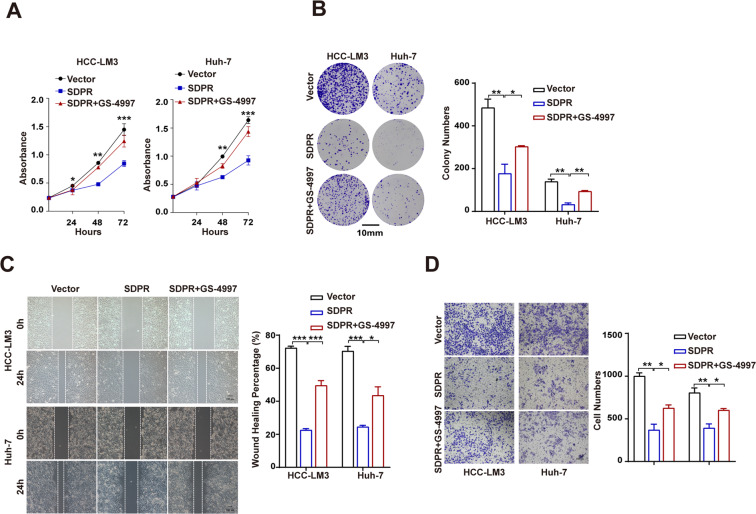
GS-4997 reversed the anti-tumor effects induced by SDPR overexpression. **A**, **B** CCK-8 assays and colony formation assays revealed that GS-4997 rescued HCC cells growth ability in SDPR overexpressing HCCLM3 and Huh-7 cell lines. **C**, **D** GS-4997 treatment significantly restored the effectiveness of SDPR overexpression on cell invasion and cell migration by transwell and wound-healing assays. *p < 0.05; ***p < 0.01; ***p < 0.001.

Based on SDPR and ASK1 coordinately inducing HCC cells apoptosis and SDPR modulating the activation of ASK1, we hypothesized that the antitumor effects of SDPR might be mediated by ASK1. Accordingly, ASK1 inhibitor GS-4997 was utilized in HCC cells biological behavior experiments. We observed that the number of proliferating cells were much more in SDPR overexpressing cells after treatment with GS-4997 (Fig. 7A, B). Furthermore, the anti-tumor phenotypes induced by SDPR overexpression were reversed by GS-4997 treatment (Fig. 7C, D). These results illustrated that the antitumor potentials of SDPR were facilitated by ASK1.

## Discussion

It is evident from the previous studies that SDPR served as an antitumor molecule in breast and gastric cancer^9,10^. Although previous study has identified decreased SDPR expression in HCC, how SDPR affected HCC progress and the underlying mechanisms were generally undiscovered. After discovering the differential expression of SDPR in tumor and non-tumor tissues, we speculated that SDPR might have an antitumor potential in HCC. Subsequently, we detected the relationship between SDPR expression with clinical characteristics and prognostication. The results demonstrated that SDPR was considerably linked with tumor size, histologic grade, PVTT, and TNM stage. Next, the phenotypic assays demonstrated that SDPR exerted significantly inhibiting effects on cell migration, invasion, and growth both in vivo and in vitro. The mechanisms of SDPR in lung cancer and breast cancer have been well studied, a study proved that SDPR might inhibit NSCLC cells malignant behaviors through p38 MAPK/ERK signals^17^, another research demonstrated that SDPR served as an apoptosis inducer thereby producing inhibitory effects on metastasis in breast tumor^9^. Then GSEA was conducted to further evaluate the mechanism of SDPR in HCC, the results identified positive enrichment of the apoptosis-related gene signature between the low and high heterogeneity groups. Accumulating with apoptosis assay, we reasoned that HCC progress inhibition might be leaded by activating cell apoptosis. What is more, SDPR significantly facilitated OXA-induced apoptosis. Previous works indicated that those agents which can directly trigger apoptosis pathways could also activate cell death pathways^18^. We hypothesized that SDPR might combine and interact with apoptosis pathway-related proteins. To confirm this, we detected SDPR-binding proteins by IP-MS assay and 259 proteins were discovered, among which Apoptosis signal-regulating kinase 1 (ASK1) aroused our attention.

ASK1, has also been identified as mitogen activated-protein kinase 5 (MAP3K5), which is stimulated by a series of stimuli such as calcium influx, endoplasmic reticulum (ER) stress, reactive oxygen species (ROS), and extracellular inflammatory signals including lipopolysaccharides (LPS) and tumor necrosis factor (ROS)^19–21^. The downstream cascade is subsequently initiated by activation of ASK1 which phosphorylates and activates two MAP2K pathways including MEE-4/7 and MKK-3/6, which thereby phosphorylates and activates c-Jun N-terminal kinase (JNK) and p38 MAPKs, respectively^22^. The basic roles of p38 and JNK MAPKs in cancer includes cell proliferation, differentiation, and apoptosis^23^. A study performed by Nakagawa indicated that the knockout of ASK1 inhibited HCC cells apoptosis apparently and promoted the development of HCC^24^. Another such study reported a key role of ASK1 in the event of HCC cell’s apoptosis, induced by sorafenib^25^. Therefore, it is extremely important to regulate the activity of ASK1 which in turn provides new insight into an anti-tumor strategy in HCC.

As a crucial mediator of pro-apoptotic signals, the activation and inhibition of ASK1 are regulated by multiple mechanisms among which protein–protein interactions (PPIs) play an important role^20^, proteins can affect ASK1 activity by combining and interacting with ASK1 N-terminal^26^. Thioredoxin (TRX) considerably inhibits ASK1 activity, particularly when it binds to ASK1 in its reduced form. However, in ROS condition TRX is converted to oxidized form and separated from ASK1. TNF receptor-associated factor 2 and 6 (TRAF2/6) have been appeared to promote ASK1 activation and homodimerization by subsequently binding to ASK1^27,28^. In addition, there is a CIBI binding region at the N-terminal of ASK1 to detect Ca^2+^ stress signals, and more protein–protein interaction sites are being detected^29^. Among with our IP-MS results, we speculated that SDPR induced apoptosis through ASK1. Therefore, further PPIs experiments were performed to confirm SDPR-ASK1 interaction and the results revealed that SDPR bound to the NCC domain of ASK1 and facilitated ASK1 N-terminus-mediated dimerization and phosphorylation activation.

**Fig. 8:**
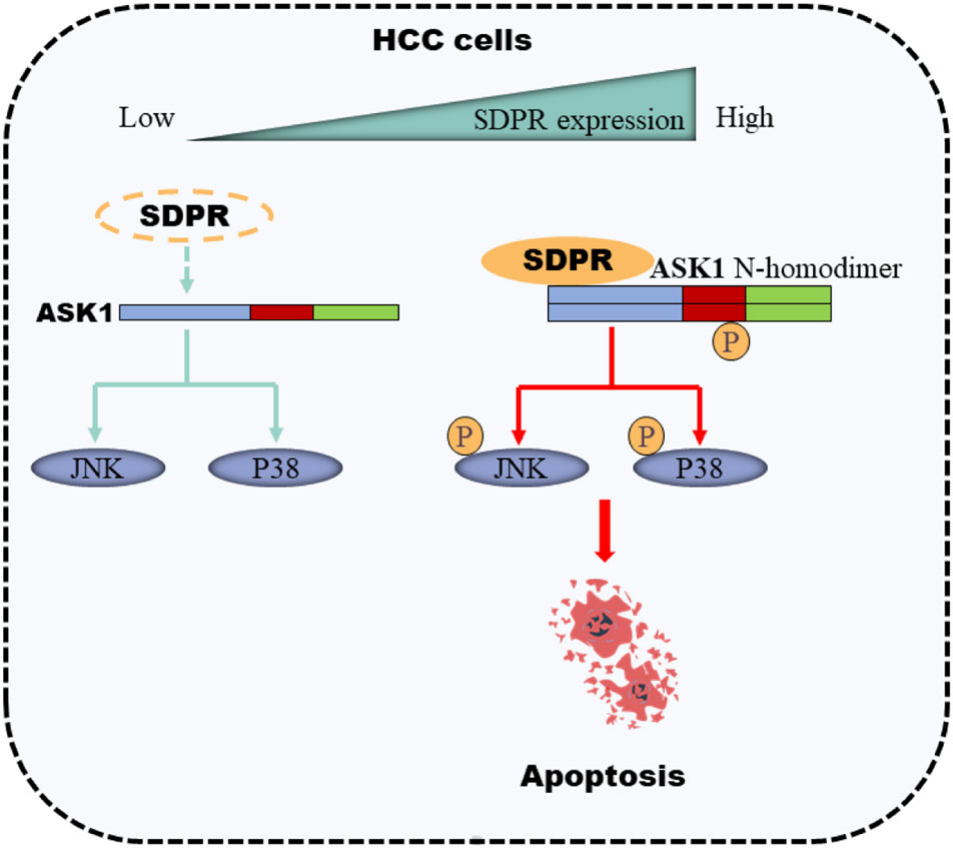
A graphical model for SDPR-mediated apoptosis in HCC cells. SDPR interacted with ASK1 to facilitate ASK1 N-terminus-mediated dimerization and increased ASK1-mediated signaling to activate the JNK/p38 MAPKs and promote cell apoptosis in HCC with SDPR high expression.

As the downstream kinases of MAP2Ks and MAP3Ks, JNK and P38 signaling pathways are selectively triggered by ASK1 and are recognized as major regulators in activating cell apoptosis^30,31^. Even so, JNK and P38 MAPKs might be triggered by other MAP3Ks including TAK1, MLK, MEKK, and so on^32^. Thus, we utilized GS-4997, which binds to the ASK1 kinase domain and thereby inhibiting ASK1 selectively^33^, to assess the changes of p-JNK and p-P38. The results indicated that GS-4997 inhibited phosphorylation of JNK/P38 and cell apoptosis induced by SDPR, to a large extent, but not all. Consequently, we concluded that SDPR induced apoptosis primarily through ASK1-JNK/P38 pathways. Considering that GS-4997 did not totally block the pro-apoptosis effect of SDPR, the cross talk between SDPR and other apoptosis pathways was still needed further exploration. Mechanically, in HCC patients with high SDPR expression, SDPR combined with ASK1 NCC and facilitated N-terminus-mediated dimerization, subsequently ASK1 was phosphorylated and activated. Followed JNK/P38 pathways were activated and induced cells apoptosis. However, these pathways were largely blocked in patients with low SDPR expression (Fig. 8).

In conclusion, our findings supported the speculation that SDPR was a tumor suppressor, which inhibited cell invasion, proliferation, and migration by inducing apoptosis in HCC. What is more, SDPR overexpression could enhance the chemotherapeutic reactivity of HCC cells to OXA. Besides, we have identified a novel PPI that SDPR combined with and activated ASK1 and then leaded to activation of the JNK/P38 MAPK pathways. The finding provided a novel antitumor mechanism that may develop effective treatment strategies of HCC.

## Supplementary information

Certificate of STR Analysis for huh7

Certificate of STR Analysis for L02

Certificate of STR Analysis for SK-Hep1

supplementary table S1

supplementary table S2

supplementary table S3

## Data Availability

The data that support the findings of this study are available from the corresponding author upon reasonable request.
