# Supplementary material for: Serum deprivation-response protein induces apoptosis in hepatocellular carcinoma through ASK1-JNK/p38 MAPK pathways
Source: Cell Death Dis. 2021 Apr 30;12(5):425. doi: 10.1038/s41419-021-03711-x (PMC8087765; doi:10.1038/s41419-021-03711-x)
Supplement: Supplementary file 1 — Certificate of STR Analysis for huh7 [file 41419_2021_3711_MOESM1_ESM.pdf]

上海艾博思生物科技有限公司
**Shanghai Integrated Biotech Solutions Co.,Ltd**
地址：上海浦东张江高科技园区紫薇路 750 弄 10 号 202 室
邮编：201203　电话：021-3877863　传真：021-38770863
E-mail:service@ibsbio.com　http://www.ibsbio.com

# Cell Line Authentication Report

Customer:

Institution:

Quotation Number: WR1608031

Completion Date：08/11/2016

1. **Sample ID: huh7**
2. **Original Material: Cell pellets**
3. **Methods:**

1).Genomic DNA was extracted from the cell pellets provided by the customer.

2).Samples, together with positive and negative control were amplified using GenePrint 10 System(Promega).

3). Amplified products were processed using the ABI3730xl Genetic Analyzer.

4).Data were analyzed using GeneMapper4.0 software and then compared with the ATCC, DSMZ or JCRB databases for reference matching.

4. **Results:**

1) **10 Loci STR Profile:**

<table>
<tr><td>Genetic Site</td><td colspan="2">ATCC</td><td colspan="3">Customer sample</td></tr>
<tr><td>(Locus)</td><td colspan="2">huh7</td><td colspan="3">huh7</td></tr>
<tr><td>Amelogenin</td><td>X</td><td></td><td colspan="2">X</td><td></td></tr>
<tr><td>CSF1PO</td><td>11</td><td></td><td colspan="2">11</td><td>12</td></tr>
<tr><td>D13S317</td><td>10</td><td></td><td>11</td><td>12</td><td>13</td></tr>
<tr><td>D16S539</td><td>10</td><td></td><td colspan="2">9</td><td>14</td></tr>
<tr><td>D5S818</td><td>12</td><td></td><td colspan="2">8</td><td>9</td></tr>
<tr><td>D7S820</td><td>11</td><td></td><td>10</td><td>11</td><td>12</td></tr>
<tr><td>THO1</td><td>7</td><td></td><td colspan="2">7</td><td>9.3</td></tr>
<tr><td>TPOX</td><td>8</td><td>11</td><td colspan="2">11</td><td></td></tr>
<tr><td>vWA</td><td>16</td><td>18</td><td>16</td><td>19</td><td>21</td></tr>
</table>

[键入文字]

| | | | | |
|---|---|---|---|---|
| D21S11 | 30 | | 28 | 29.2 |
| Percent match between the sample and the database profile：36.4% | | | | |

Summary:

1) Your cell linedid not match the reference cell line in the ATCC STR database,as the STR profile yields matches that are less than 80%.

Notes：

1. P=100% x (2xM)/N; M=6, N=33 P=100% x (2x6)/33=36.4%

M: number of the matching peaks;　N: number of all peaks

2. Based on the ANSI Standard, cell lines with ≥80% match are considered to be related; i.e.,derived from a common ancestry. Cell lines with between a 55% to 80% match require futher profiling for authentication of relatedness.

3. The short tandem repeat (STR) profile generated by GENEWIZ Inc. is indicative only of the sample sent to GENEWIZ Inc. at the time it was sent. This data and analysis are for research use only.

<<<If the Percent match is not 100%, search for reference matchingwith the ATCC, DSMZ or JCRB databases and add the match results.

Addendum: Comparative output from the ATCC STR Profile database

## Result of STR matching analysis by your data.

- DSMZ Profile Database -

A graphical presentation is shown at the bottom of this page.

| EV | Cell No. | Cell name | Locus names | | | | | | | | | Figures |
|---|---|---|---|---|---|---|---|---|---|---|---|---|
| | | | D5S818 | D13S317 | D7S820 | D16S539 | VWA | TH01 | AM | TPOX | CSF1PO | |
| | Query (Your Cell) | | 8, 9 | 11, 12, 13 | 10, 11, 12 | 9, 14 | 16, 19, 21 | 7, 9.3 | X | 11 | 11, 12 | |
| 0.92(34/37) | 305 | 293 | 8, 9 | 12, 14 | 11, 12 | 9, 9 | 16, 19 | 7, 9.3 | X, X | 11, 11 | 11, 12 | - |
| 0.92(34/37) | CRL-10852 | 293 c18 | 8, 9 | 12, 14 | 11, 12 | 9, 9 | 16, 19 | 7, 9.3 | X, X | 11, 11 | 11, 12 | - |
| 0.92(34/37) | CRL-11654 | 90, 74 | 8, 9 | 12, 14 | 11, 12 | 9, 9 | 16, 19 | 7, 9.3 | X, X | 11, 11 | 11, 12 | - |
| 0.92(34/37) | CRL-12007 | ProPak-X.36 [PP-X.36] | 8, 9 | 12, 14 | 11, 12 | 9, 9 | 16, 19 | 7, 9.3 | X, X | 11, 11 | 11, 12 | - |
| 0.92(34/37) | CRL-12013 | 2A | 8, 9 | 12, 14 | 11, 11 | 9, 9 | 16, 19 | 7, 9.3 | X, X | 11, 11 | 12, 12 | - |
| 0.92(34/37) | CRL-12386 | SODK1 | 8, 9 | 12, 14 | 11, 12 | 9, 9 | 16, 19 | 7, 9.3 | X, X | 11, 11 | 11, 12 | - |
| 0.92(34/37) | CRL-12479 | ProPak-A.52 Clone #52 [PP-A.52] | 8, 9 | 12, 14 | 11, 12 | 9, 9 | 16, 19 | 7, 9.3 | X, X | 11, 11 | 11, 12 | - |
| 0.92(34/37) | CRL-2828 | PEAKrapid | 8, 9 | 12, 14 | 11, 12 | 9, 9 | 16, 19 | 7, 9.3 | X, X | 11, 11 | 11, 12 | - |
| 0.92(34/37) | CRL-3022 | HEK 293S GnTI- | 8, 9 | 12, 14 | 11, 12 | 9, 9 | 16, 19 | 7, 9.3 | X, X | 11, 11 | 11, 12 | - |
| 0.92(34/37) | JCRB9068 | 293 | 8, 9 | 12, 12 | 11, 12 | 9, 13 | 16, 19 | 7, 9.3 | X, X | 11, 11 | 11, 12 | - |
| 0.92(34/37) | RCB2253 | HKb20 | 8, 9 | 12, 12 | 11, 11 | 9, 13 | 16, 19 | 7, 9.3 | X, X | 11, 11 | 11, 12 | - |
| 0.92(34/37) | RCB2354 | 293gp | 8, 9 | 12, 14 | 11, 12 | 9, 9 | 16, 19 | 7, 9.3 | X, X | 11, 11 | 11, 12 | - |

>>>

## 2) Electrophoretogram

Applied Biosystems
GeneMapper 4.0

M10128

| Sample File | Sample Name | Panel | SQI | OS | SQ |
|---|---|---|---|---|---|
| huh7_G12.fsa | huh7 | GenePrint_10_v1.1 | | | |

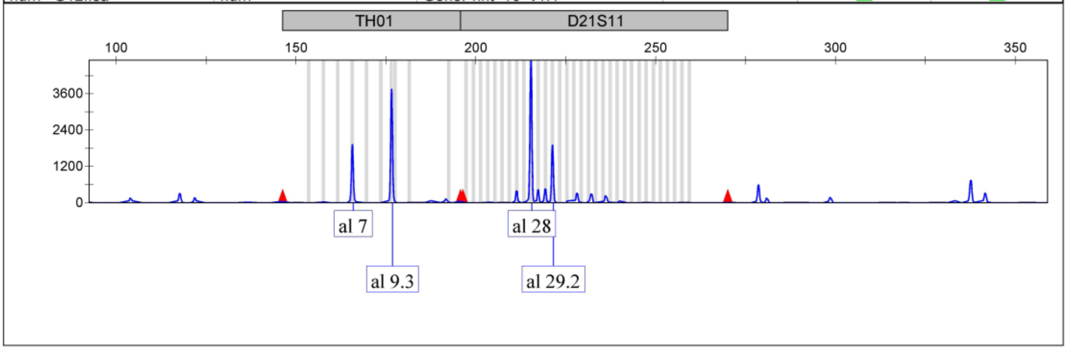


| | | | | | |
|---|---|---|---|---|---|
| huh7_G12.fsa | huh7 | GenePrint_10_v1.1 | | | |

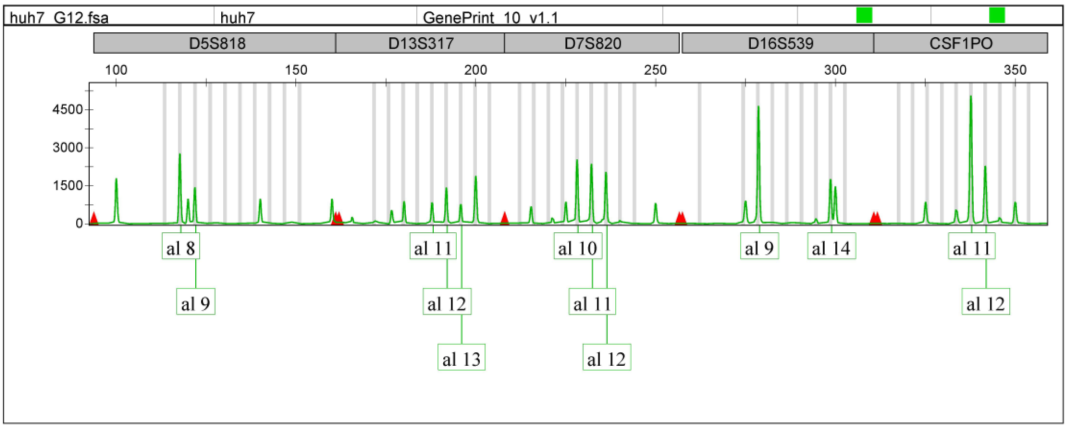


| | | | | | |
|---|---|---|---|---|---|
| huh7_G12.fsa | huh7 | GenePrint_10_v1.1 | | | |

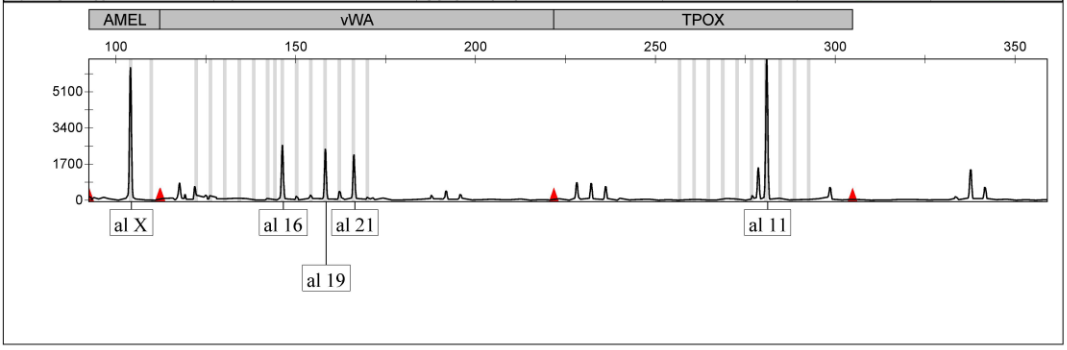


Tue Aug 09,2016 03:47PM, CST　　Printed by: gm　　Page 1 of 1

Note：Raw data in appendix

上海艾博思生物科技有限公司
**Shanghai Integrated Biotech Solutions Co.,Ltd**
地址：上海浦东张江高科技园区紫薇路 750 弄 10 号 202 室
邮编：201203　电话：021-3877863　传真：021-38770863
E-mail:service@ibsbio.com　http://www.ibsbio.com
