# Supplementary material for: Serum deprivation-response protein induces apoptosis in hepatocellular carcinoma through ASK1-JNK/p38 MAPK pathways
Source: Cell Death Dis. 2021 Apr 30;12(5):425. doi: 10.1038/s41419-021-03711-x (PMC8087765; doi:10.1038/s41419-021-03711-x)
Supplement: Supplementary file 2 — Certificate of STR Analysis for L02 [file 41419_2021_3711_MOESM2_ESM.pdf]

表 1：样本 Lo2 的 STR 位点和 Amelogenin 位点的基因分型结果

Table 1: STR profiles of Sample

	**Sample**<br>Lo2	**Source: PubMed=26116706;**<br>LO2,94% Match
**Marker**	**Allele**	**Allele**
D3S1358	15,18	
D5S818	11,12	11,12
D2S1338	17	
TPOX	12	12
CSF1PO	10	10
Penta D	8,15	
Indel		
AMEL	X	X
TH01	7	7
vWA	16	16,18
D7S820	12	12
D21S11	27,28	
Penta E	7,17	
D10S1248	13	
D8S1179	12	
D1S1656	12,15	
D18S51	16	
D12S391	20	
D6S1043	18	
D19S433	13	
D16S539	9,10	9,10
D13S317	13.3	13.3
FGA	18,21	

Sample File	Sample Name	Panel	SQO	SOS	SQ	SSPK	MIX	OMR	CGQ
2019-CELL-00439-L3_E06.fsa	Lo2	SifaSTR 23 plex_v5.0a							

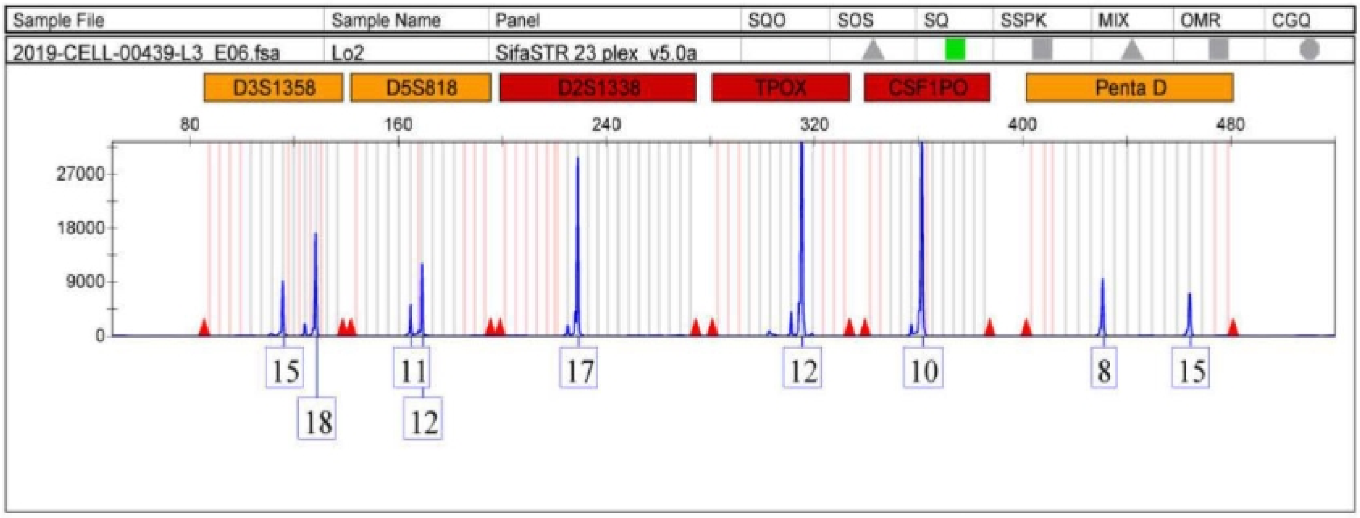

2019-CELL-00439-L3_E06.fsa	Lo2	SifaSTR 23 plex_v5.0a

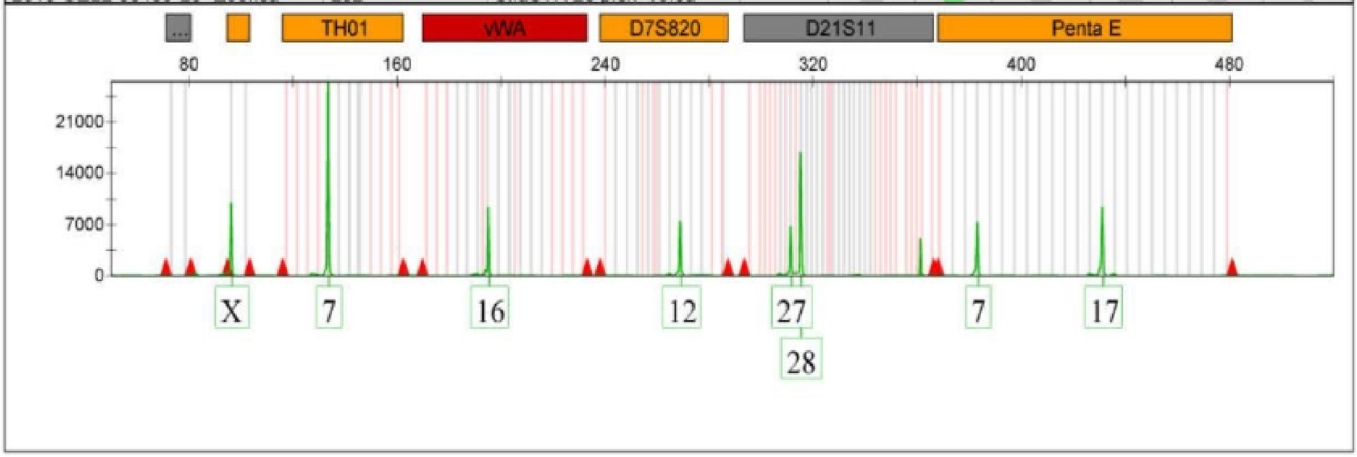

2019-CELL-00439-L3_E06.fsa	Lo2	SifaSTR 23 plex_v5.0a

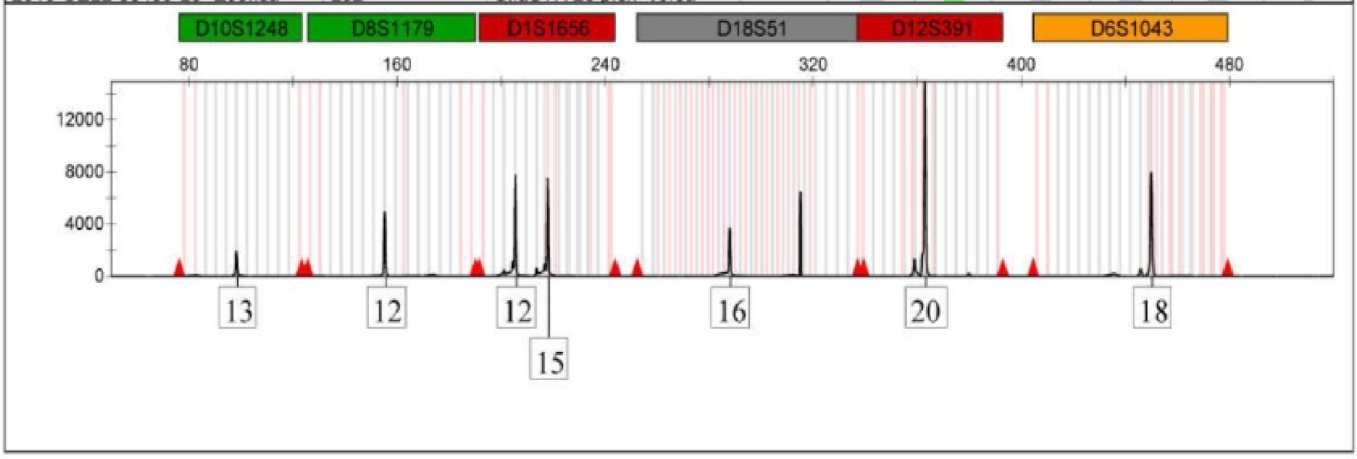

2019-CELL-00439-L3_E06.fsa	Lo2	SifaSTR 23 plex_v5.0a

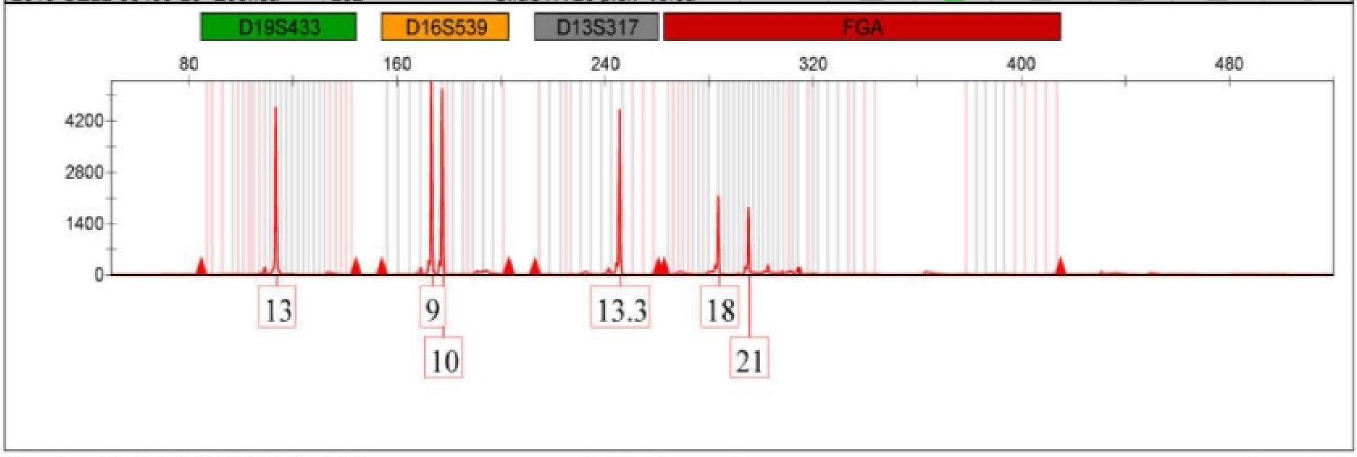

<table>
<tr><td>Cell line name</td><td>L-02</td></tr>
<tr><td>Synonyms</td><td>L02; LO2; HL-7702; HL7702; Liver-02; Human Liver-7702</td></tr>
<tr><td>Accession</td><td>CVCL_6926</td></tr>
<tr><td>Resource Identification Initiative</td><td>To cite this cell line use: L-02 (RRID:CVCL_6926)</td></tr>
<tr><td>Comments</td><td>Problematic cell line: Contaminated. Shown to be a HeLa derivative (PubMed=26116706). Originally thought to originate from a normal fetal liver.
Transformant: NCBI_TaxID; 333761; Human papillomavirus type 18 (HPV18).
Omics: Proteome analysis.
Omics: SNP array analysis.
Omics: Transcriptome analysis.</td></tr>
<tr><td>Disease</td><td>Human papillomavirus-related endocervical adenocarcinoma (NCIt: C27677)</td></tr>
<tr><td>Species of origin</td><td>Homo sapiens (Human) (NCBI Taxonomy: 9606)</td></tr>
<tr><td>Hierarchy</td><td>Parent: CVCL_0030 (HeLa)
Children:
CVCL_WN40 (HL-7702BaPT)</td></tr>
<tr><td>Sex of cell</td><td>Female</td></tr>
<tr><td>Age at sampling</td><td>30Y6M</td></tr>
<tr><td>Category</td><td>Cancer cell line</td></tr>
<tr><td>STR profile</td><td>Source(s): PubMed=26116706

Markers:
<table>
<tr><td>Amelogenin</td><td>X</td></tr>
<tr><td>CSF1PO</td><td>10</td></tr>
<tr><td>D3S1358</td><td>15,18</td></tr>
<tr><td>D5S818</td><td>11,12</td></tr>
<tr><td>D7S820</td><td>12</td></tr>
<tr><td>D8S1179</td><td>12</td></tr>
<tr><td>D13S317</td><td>13.3</td></tr>
<tr><td>D16S539</td><td>9,10</td></tr>
<tr><td>D18S51</td><td>16</td></tr>
<tr><td>D21S11</td><td>27,28</td></tr>
<tr><td>FGA</td><td>18,21</td></tr>
<tr><td>Penta D</td><td>8,15</td></tr>
<tr><td>Penta E</td><td>7,17</td></tr>
<tr><td>TH01</td><td>7</td></tr>
<tr><td>TPOX</td><td>12</td></tr>
<tr><td>vWA</td><td>16,18</td></tr>
</table>
Run an STR similarity search on this cell line</td></tr>
</table>
