# Supplementary material for: Serum deprivation-response protein induces apoptosis in hepatocellular carcinoma through ASK1-JNK/p38 MAPK pathways
Source: Cell Death Dis. 2021 Apr 30;12(5):425. doi: 10.1038/s41419-021-03711-x (PMC8087765; doi:10.1038/s41419-021-03711-x)
Supplement: Supplementary file 3 — Certificate of STR Analysis for SK-Hep1 [file 41419_2021_3711_MOESM3_ESM.pdf]

CBTCCCA

Certificate of STR Analysis

1.Sample

SK-HEP-1

2.2.Methods

The genomic DNA was purified with Purelink@ Genomic DNA Kits in our Bank.

The DNA sample was analysed in Beijing Microread GeneticsCo.,Ltd.

The sample was amplified with Goldeneye™20A STR Complex Amplification Kit.

The profiles STR loci and Amelogenin gene were characterized on ABI 3100 Type Genetic Analysis Instrument.

3.Results

D5S818	10,13
D13S317	8,12
D7S820	8,11
D16S539	12
vWA	14,17
TH01	7,9
Amelogenin	X
TPOX	9
CSF1PO	11,12

The above results were consistent with the DNA profiles reported by ATCC,and DSMZ, and indicated no other human cell lines contamination.

Cell Bank,
Type Culture Collection,
Chinese Academy of Sciences
(CBTCCCAS)

2013/10/17
