# Supplementary material for: Serum deprivation-response protein induces apoptosis in hepatocellular carcinoma through ASK1-JNK/p38 MAPK pathways
Source: Cell Death Dis. 2021 Apr 30;12(5):425. doi: 10.1038/s41419-021-03711-x (PMC8087765; doi:10.1038/s41419-021-03711-x)
Supplement: Supplementary file 4 — supplementary table S1 [file 41419_2021_3711_MOESM4_ESM.docx]

**Supplementary Table S1. Sequences of primers used for PCR in this study**

| Primer names | sequences |
| --- | --- |
| GAPDH forward | 5’-GGAGCGAGATCCCTCCAAAAT-3’ |
| GAPDH reverse | 5’-GGCTGTTGTCATACTTCTCATGG-3’ |
| SDPR forward | 5’-AAGAGCGCATGGATAGGCAG-3’ |
| SDPR reverse | 5’-TCATCGTGGGGCAAATCATCA-3’ |
