# Supplementary material for: Serum deprivation-response protein induces apoptosis in hepatocellular carcinoma through ASK1-JNK/p38 MAPK pathways
Source: Cell Death Dis. 2021 Apr 30;12(5):425. doi: 10.1038/s41419-021-03711-x (PMC8087765; doi:10.1038/s41419-021-03711-x)
Supplement: Supplementary file 5 — supplementary table S2 [file 41419_2021_3711_MOESM5_ESM.docx]

**Supplementary Table S2. Primary antibodies used in this study**

| Antigens | Manufacturer | Catalog Number | Application |
| --- | --- | --- | --- |
| SDPR | Abcam | ab76867 | 1:750 for WB |
| SDPR | Proteintech | 12339-1-AP | 1:100 for IHC |
| GAPDH | Proteintech | 60004-1-Ig | 1:10000 for WB |
| Vimentin | Abcam | ab92547 | 1:1000 for WB; 1:500 for IHC |
| E-cadherin | Abcam | ab231303 | 1:1000 for WB; 1:500 for IHC |
| N-cadherin | Abcam | ab76057 | 1:1000 for WB; 1:500 for IHC |
| Bax | Cell signaling technology | #2772 | 1:1000 for WB |
| BCL-2 | Cell signaling technology | #2870 | 1:1000 for WB |
| Cleaved- Caspase3 | Cell signaling technology | #9664 | 1:1000 for WB |
| Caspase3 | Cell signaling technology | #9662 | 1:1000 for WB |
| Cleaved-PARP | Abcam | ab191909 | 1:1000 for WB |
| PARP | Cell signaling technology | #9532 | 1:1000 for WB |
| FLAG-tag | MBL | M185-3LL | 1:10000 for WB; 1:1000 for IF; 1:500 for IP |
| HA-tag | Cell signaling technology | #3724 | 1:1000 for WB; 1:800 for IF; 1:500 for IP |
| MYC-tag | MBL | M047-3 | 1:1000 for WB |
| ASK1 | Abcam | ab45178 | 1:2000 for WB |
| p-ASK1 | Cell signaling technology | #3765 | 1:1000 for WB |
| ERK | Cell signaling technology | #4695 | 1:1000 for WB |
| p-ERK | Cell signaling technology | #4370 | 1:1000 for WB |
| JNK1/2 | Cell signaling technology | #9252 | 1:1000 for WB |
| p-JNK1/2 | Cell signaling technology | #4668 | 1:1000 for WB |
| P38 | Cell signaling technology | #9212 | 1:1000 for WB |
| p-P38 | Cell signaling technology | #4511 | 1:1000 for WB |
