# Supplementary material for: Serum deprivation-response protein induces apoptosis in hepatocellular carcinoma through ASK1-JNK/p38 MAPK pathways
Source: Cell Death Dis. 2021 Apr 30;12(5):425. doi: 10.1038/s41419-021-03711-x (PMC8087765; doi:10.1038/s41419-021-03711-x)
Supplement: Supplementary file 6 — supplementary table S3 [file 41419_2021_3711_MOESM6_ESM.docx]

**Supplementary Table S3. Sequences of siRNA against SDPR in this study**

| Names | sequences |
| --- | --- |
| siRNA NC | 5’-UUCUCCGAACGUGUCACGUTT-3’ |
| siRNA #1 | 5’-GCATCCAGAATGACCTCACCA-3’ |
| siRNA #2 | 5’-GGCACAGAAGGTACGCTATGA-3’ |
| siRNA #3 | 5’-CGACTTGACTATTGTGGAA-3’ |
